# A Vest-Pocket Medical Lexicon

**Published:** 1867-03

**Authors:** 


A Vest-Pocket Medical Lexicon: Being a Dictionary of the Words, Terms, and Symbols of Medical Science. Collated From the Best Authorities, with the Addition of New Words not before introduced into a Lexicon. With an Appendix. By D. B. ST. JOHN ROOSA, M.D., Prof. Aural Surgery, etc. New York: WM. WOOD & Co., 61 Walker Street. 1867.

This is just what its title-page would indicate, namely, a very neat and convenient medical dictionary, so small that the student can carry it in his pocket, with perfect ease; and so cheap as to be within the reach of all. It indicates the accent, or pronunciation, and the direct definition of a large number of words, though in so small a compass.

